# Exogenous Heparan Sulfate Enhances the TGF-*β*3-Induced Chondrogenesis in Human Mesenchymal Stem Cells by Activating TGF-*β*/Smad Signaling

**DOI:** 10.1155/2016/1520136

**Published:** 2015-12-13

**Authors:** Juan Chen, Yongqian Wang, Chong Chen, Chengjie Lian, Taifeng Zhou, Bo Gao, Zizhao Wu, Caixia Xu

**Affiliations:** ^1^Research Center for Translational Medicine, The First Affiliated Hospital of Sun Yat-sen University, Guangzhou 510080, China; ^2^School of Life Sciences, Sun Yat-sen University, Guangzhou 510275, China; ^3^Department of Musculoskeletal Oncology, The First Affiliated Hospital of Sun Yat-sen University, Guangzhou 510080, China; ^4^Department of Orthopedic Surgery, The First Affiliated Hospital of Sun Yat-sen University, Guangzhou 510080, China; ^5^Department of Spine Surgery, Sun Yat-sen Memorial Hospital, Guangzhou 510120, China

## Abstract

Heparan sulfate (HS) interacts with growth factors and has been implicated in regulating chondrogenesis. However, the effect of HS on TGF-*β*-mediated mesenchymal stem cell (MSC) chondrogenesis and molecular mechanisms remains unknown. In this study, we explored the effects of exogenous HS alone and in combination with TGF-*β*3 on chondrogenic differentiation of human MSCs and possible signal mechanisms. The results indicated that HS alone had no obvious effects on chondrogenic differentiation of human MSCs and TGF-*β*/Smad2/3 signal pathways. However, the combined TGF-*β*3/HS treatment resulted in a significant increase in GAG synthesis, cartilage matrix protein secretion, and cartilage-specific gene expression compared to cells treated with TGF-*β*3 alone. Furthermore, HS inhibited type III TGF-*β* receptors (T*β*RIII) expression and increased TGF-*β*3-mediated ratio of the type II (T*β*RII) to the type I (T*β*RI) TGF-*β* receptors and phosphorylation levels of Smad2/3. The inhibitor of the TGF-*β*/Smad signal, SB431542, not only completely inhibited HS-stimulated TGF-*β*3-mediated Smad2/3 phosphorylation but also completely inhibited the effects of HS on TGF-*β*3-induced chondrogenic differentiation. These results demonstrate exogenous HS enhances TGF-*β*3-induced chondrogenic differentiation of human MSCs by activating TGF-*β*/Smad2/3 signaling.

## 1. Introduction

Mesenchymal stem cells (MSCs), because of their extensive proliferative capacity and strong chondrogenic potential, represent a promising cell source for cartilage repair [1, 2]. Effective chondrogenic induction of MSCs for the repair of cartilage damage remains a great challenge. A lot of research has focused on the factors and molecular mechanisms enhancing chondrogenic potential of MSCs [3, 4]. Among the factors, growth factors play important roles in regulating chondrogenesis [5]. Transforming growth factor-*β*3 (TGF-*β*3), a member of TGF-*β* superfamily, is the most extensively used growth factor for inducing differentiation of MSCs [6]. Studies have demonstrated that TGF-*β*3 stimulates cartilage formation both in vitro and in vivo, producing more collagen II and aggrecan in MSC cultures than either TGF-*β*1 or TGF-*β*2 [5, 7].

TGF-*β* enhances the expression of chondrogenic markers by activating typical TGF-*β*/Smad signaling. TGF-*β* signaling is initiated through the sequential activation of two serine/threonine kinase receptors: the type II (T*β*RII) and the type I (T*β*RI) TGF-*β* receptors. The TGF-*β* ligand, binding to T*β*RII, phosphorylates and activates T*β*RI to form a large ligand-receptor complex, which then activates downstream Smad2/3 molecule and induce TGF-*β*-dependent transcriptional programs [8]. The type III TGF-*β* receptor (T*β*RIII), also named betaglycan, is a widely expressed heparan sulfate (HS) and chondroitin sulfate (CS) proteoglycan that is believed to be a coreceptor for TGF-*β* [9]. T*β*RIII modulates TGF-*β* signaling by binding and presenting TGF-*β*s ligand to T*β*RII [10, 11].

Recently, there is increasing evidence that extracellular matrix (ECM), as a major component of cell niche, plays a central role in MSCs proliferation and differentiation through the regulation of the growth factor interactions between the ECM and cells [12, 13]. Heparan sulfates (HSs) are highly sulfated glycosaminoglycans (GAGs) [14, 15]. In vivo, they covalently attach to different core proteins to form heparan sulfate proteoglycans (HSPGs), which exist on the cell surfaces or in the ECM of multiple tissues, including developing and mature cartilage [16, 17]. Studies have demonstrated that HSPGs play key roles in cartilage development and skeletal growth [18–20]. HS chains in HSPG interactions with a variety of chondroregulatory molecules have been implicated in regulating chondrogenesis through various signaling pathways, including fibroblast growth factors (FGFs), bone morphogenetic proteins (BMPs), TGF-*β*s, Wnt, and Hedgehog [20]. TGF-*β*s are important chondroregulatory factors that have been shown to interact with HS [21]. Chen et al. reported that HSPGs might play an important role in regulating TGF-*β* through the regulation of latent transforming growth factor-*β*-binding protein (LTBP1) assemblies [22]. Cell-surface HS proteoglycans have been shown to modulate TGF-*β* responsiveness in epithelial cells and other cell types [10]. However, the direct role of exogenous HS in TGF-*β*-mediated chondrogenesis of MSCs and corresponding molecular mechanisms remains to be demonstrated.

We used an in vitro human MSC (hMSC) chondrogenic differentiation model to study the role of exogenous HS in TGF-*β*3-induced chondrogenesis and TGF-*β*/Smad signaling. Our results suggest that exogenous HS clearly potentiates TGF-*β*3-induced chondrogenic differentiation of hMSCs by modulating the expression mode of TGF-*β* receptors and by activating the downstream Smad signaling pathway.

## 2. Materials and Methods

### 2.1. Cell Isolation and Culture

The Ethics Committee of the First Affiliated Hospital of Sun Yat-sen University approved this study, and all the subjects provided written informed consent. The bone marrow samples are from three healthy volunteer donors with an age range of 18 to 22 years. They have no physical disease. hMSCs were isolated and purified by the following method of density gradient centrifugation [23]. Briefly, the bone marrow samples were added to Ficoll-Paque (1.077 g/mL) (TBD, Tianjin, China) and centrifuged for 20 min at 500 g. The mononuclear cells were resuspended in low-glucose Dulbecco's modified Eagle medium (L-DMEM) (Gibco, Invitrogen Corporation, NY, USA) supplemented with 10% fetal bovine serum (FBS) (Gibco, Invitrogen Corporation, Uruguay) and were incubated at 37°C under 5% CO_2_. After 48 h, nonadherent cells were removed by changing the medium. Cells were passaged in culture when 80–90% confluence was reached. We used cells from passage 3 to passage 6 in our experiments.

### 2.2. Chondrogenic Differentiation of hMSCs in Pellet Culture

Human MSCs were harvested and resuspended at 2 × 10^7^ cells/mL, according to the following procedure [24]. Cell droplets (4 × 10^5^/20 *μ*L) were divided into four groups. Group 1 (C group) was maintained in the chondrogenic control medium consisting of high-glucose DMEM (H-DMEM), supplemented with 50 *μ*g/mL vitamin C, 100 nM dexamethasone, 1 mM sodium pyruvate, 40 *μ*g/mL proline, and ITS+ Universal Culture Supplement Premix (BD Biosciences, NY, USA) (final concentrations: 6.25 *μ*g/mL bovine insulin, 6.25 *μ*g/mL transferrin, 6.25 *μ*g/mL selenous acid, 5.33 *μ*g/mL linoleic acid, and 1.25 mg/mL bovine serum albumin (BSA)). Group 2 (HS group) was maintained in the control medium, supplemented with 100 *μ*g/mL HS (Sigma-Aldrich, St. Louis, USA). Group 3 (T group) was maintained in the control medium, supplemented with 10 ng/mL TGF-*β*3 (PeproTech, Rocky Hill, USA). Group 4 (T + HS group) was maintained in the control medium, supplemented with 100 *μ*g/mL HS and 10 ng/mL TGF-*β*3. Cell droplets were incubated at 37°C/5% CO_2_. The medium was changed every 3 days, and induced cartilage tissues were harvested on days 3, 7, 14, and 21.

### 2.3. Quantitative Analysis of Glycosaminoglycan (GAG)

The harvested cartilage balls were washed and then digested in phosphate-buffered saline (PBS) solution containing 0.03% papain, 5 mM cysteine hydrochloride, and 10 mM EDTA-Na2 for 16 h at 65°C. The DNA concentration was measured using the Hoechst 33258 binding assay. Briefly, an aliquot of the lysate was reacted with 0.7 *μ*g/mL Hoechst 33258 solution (Sigma-Aldrich, St. Louis, USA) for 10 min and then was measured using a SpectraMax M5 Microplate Reader (Molecular Devices, Sunnyvale, CA, USA) at 340 nm for excitation and 465 nm for emission. The 1,9-dimethylmethylene blue (DMMB) (Sigma-Aldrich, St. Louis, USA) dye binding assay was used for detecting GAG concentration. Similarly, an aliquot of the lysate was reacted with DMMB solution for 10 min in the absence of light, and the absorbance at 525 nm was measured using a Varioskan Flash Multimode Reader (Thermo Scientific, Waltham, MA, USA). GAG content was normalized against DNA content.

### 2.4. Histology and Immunohistochemistry

The different groups of chondrogenic pellets were harvested on day 7 after chondrogenic induction. The pellets were fixed in 4% paraformaldehyde for 1 day and embedded in paraffin. Paraffin sections (4 *μ*m thick) were deparaffinized using xylene, rehydrated through a graded series of washes in ethanol, and finally rinsed in PBS. Sections were stained with hematoxylin and eosin (HE) (Sigma-Aldrich, St. Louis, USA) for cartilage structure and 0.1% Alcian blue (AB) (Sigma-Aldrich, St. Louis, USA) for proteoglycan. For immunohistochemistry, rehydrated sections were treated with a pepsin solution at 37°C for 10 min, incubated with 3% H_2_O_2_ for 10 min and with blocking serum for 15 min, and then were allowed to react overnight with rabbit anti-human collagen type II polyclonal antibodies (Abzoom Biolabs, Dallas, TX, USA), diluted at 1 : 1000 at a temperature of 4°C. Afterwards, biotinylated goat anti-rabbit IgG (EarthOx, SFO, USA) was applied for 30 min. Sections were incubated with peroxide-conjugated streptavidin working solution and stained with 3,3′-diaminobenzidine tetrahydrochloride (DAB) (Jinshan Jinqiao, Beijing, China), and staining was visualized using an Axio observer Z1 microscope (Zeiss, Göttingen, Germany).

For fluorescent immunohistochemistry staining, tissue sections were microwaved in a 10 mM citrate buffer, blocked for 1 h with PBS containing 5% BSA, and reacted overnight with the appropriate primary antibodies (human T*β*RI antibody (Santa Cruz, Dallas, USA); human T*β*RII antibody (RD Systems); and human T*β*RIII antibody (Santa Cruz, Dallas, USA)), diluted at 1 : 50 at 4°C. Tissue sections were incubated then with fluorescein isothiocyanate (FITC) conjugated secondary antibodies (diluted 1 : 100) for 1 h at room temperature. Finally, sections were stained with (4′,6-diamidino-2-phenylindole) DAPI (1 mg/mL), covered with glycerol, and examined using a Zeiss LSM 710 confocal microscope (Carl Zeiss, Heidelberg, Germany).

### 2.5. RNA Extraction and Real-Time PCR Analysis

Total RNA was extracted from pellets using an RNAsimple Total RNA Kit (Tiangen, China) and reverse transcribed into cDNA using a PrimeScript RT Reagent Kit (Takara, Osaka, Japan) at day 7 after chondrogenic induction. Real-time polymerase chain reaction (PCR) was performed in triplicate using a Bio-Rad real-time PCR Detection System with iQ 5 optical system software (Bio-Rad Laboratories, Hercules, CA, USA) and SYBR Green I Master Mix (Takara, Osaka, Japan). Expression of the following genes was analyzed: aggrecan (ACAN); collagen type II, alpha 1 (COL2A1); SRY (sex determining region Y)-box 9 (SOX9); T*β*RI; T*β*RII; and T*β*RIII. The level of expression of the glyceraldehyde-3-phosphate dehydrogenase (GAPDH) gene was used as an internal control. The primer sequences are listed in Table 1. The relative expression levels for each target gene were calculated using the 2^−ΔΔCT^ method.

### 2.6. Western Blot

After 24 h of chondrogenic induction, proteins were extracted from the pellets with radioimmunoprecipitation assay (RIPA) lysis buffer containing protease and phosphatase (CWBio, Beijing, China). The protein concentration was then measured with a bicinchoninic acid assay using a BCA Protein Assay Kit (CWBio, Beijing, PR China) and conserved at –80°C. For the western blot, equal amounts of proteins were separated by sodium dodecyl sulfate polyacrylamide gel electrophoresis and transferred onto a polyvinylidene difluoride (PVDF) membrane (Millipore, Boston, USA) at 250 mM for 100 minutes using a PowerPac Basic electrophoresis apparatus (Bio-Rad, Hercules, USA). The PVDF membranes were blocked for 1 h with 5% skim milk/Tris-buffered saline containing 0.1% Tween-20 (TBST) and then were incubated overnight at 4°C with the appropriate primary antibodies: rabbit anti-phospho-Smad2 (Ser465/467)/Smad3 (Ser423/425) (Cell Signaling), rabbit anti-Smad2/3 (Cell Signaling, Danvers, USA), and anti-GAPDH monoclonal antibody (EarthOx, SFO, USA). All the primary antibodies were applied at a 1 : 1000 dilution. After the primary antibody reaction, target proteins were detected using HRP-conjugated goat anti-rabbit IgG (diluted 1 : 10,000) for 1 h. The immune complexes were then detected using SuperSignal West Pico Chemiluminescent Substrate (Pierce, NY, USA) and they were visualized via the Image Quant Las4000mini (GE Healthcare, UK). The protein levels in the phosphorylated Smad2/3 were quantified and normalized to the total Smad2/3 quantities.

### 2.7. Inhibition of TGF-*β*/Smad Signaling

To assess the role of the TGF-*β*/Smad signaling in HS regulation of TGF-*β*3-induced hMSCs chondrogenic differentiation, the cells were treated with or without SB431542 (Sigma-Aldrich, St. Louis, USA) 2 h before stimulation by either 10 ng/mL of TGF-*β*3 alone or 100 *μ*g/mL HS combined with 10 ng/mL TGF-*β*3. SB431542 is a selective inhibitor of activin receptor-like kinase ALK5 (T*β*RI), whereas Smad2 and Smad3 are substrates for ALK5. SB431542 has been demonstrated as being the specific inhibitor for TGF-*β*/Smad pathway [25]. After 7 days of chondrogenic induction, the cells were collected, and phospho-Smad2 (Ser465/467)/Smad3 (Ser423/425) antibodies were detected by western blot. The chondrogenic differentiation ability of hMSCs was assayed by immunohistochemistry staining for collagen type II and by real-time PCR for chondrogenic genes expression.

### 2.8. Statistical Analysis

All quantitative data were presented as mean values ± standard errors (SE). Statistical analysis, consisting of one-way ANOVA followed by a LSD *t*-test, was performed using SPSS 16.0 statistical software (SPSS, Chicago, IL, USA). *P* < 0.05 was chosen as the threshold of significance.

## 3. Results

### 3.1. HS Promotes TGF-*β*3-Induced Chondrogenic Differentiation of hMSCs

**Figure 1:**
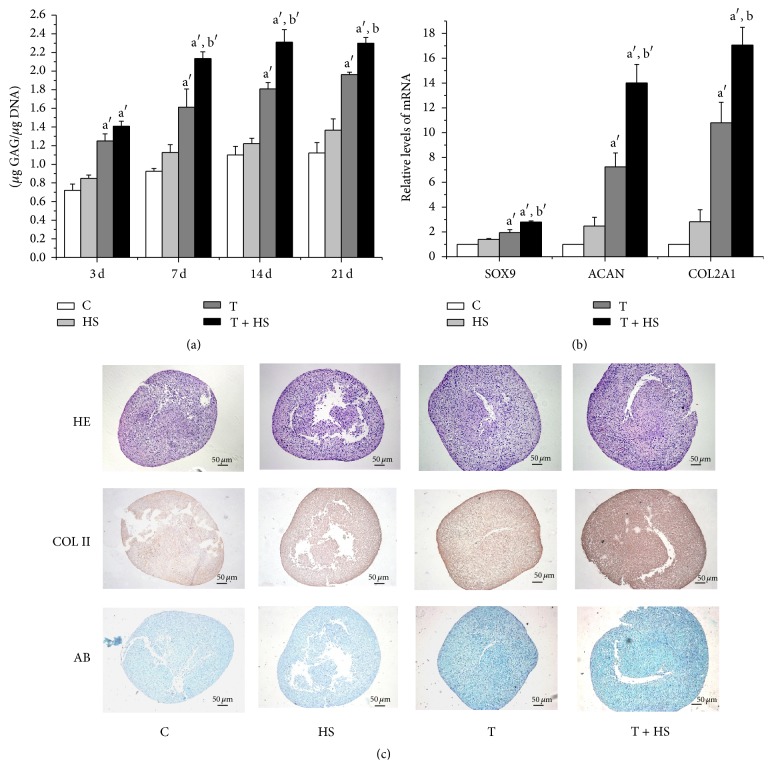
Heparan sulfate promotes TGF-*β*3-induced chondrogenic differentiation of hMSCs. Cells were cultured in control medium (C), heparan sulfate (HS), TGF-*β*3 (T), or heparan sulfate together with TGF-*β*3 (T + HS) for 21 days (*n* = 4). (a) Glycosaminoglycan (GAG) quantification at days 3, 7, 14, and 21. ^a′^ *P* < 0.01 versus C group, ^b^ *P* < 0.05 versus T group, and ^b′^ *P* < 0.01 versus T group. (b) Real-time PCR analysis of cartilage-specific genes SRY (sex determining region Y)-box 9 (SOX9), aggrecan (ACAN), and collagen type II (COL2A1) at day 7. ^a′^ *P* < 0.01 versus C group, ^b^ *P* < 0.05 versus T group, and ^b′^ *P* < 0.01 versus T group. (c) Hematoxylin and eosin (HE) staining for cartilage structure, Alcian blue staining for proteoglycan, and immunohistochemistry for collagen type II at day 7. Scale bar = 50 *μ*m.

HS alone did not elevate the synthesis of GAG greatly at different time points (Figure 1(a); *P* > 0.05). Cartilage-specific gene expression (Figure 1(b); *P* > 0.05) and proteoglycan and collagen type II secretion (Figure 1(c)) were also not elevated significantly compared with that of the untreated controls. The cells treated with TGF-*β*3 produced more GAG (Figure 1(a); *P* < 0.01) at days 3, 7, 14, and 21 and more cartilage matrix proteins (Figure 1(c)), as well as increased cartilage-specific gene expression (Figure 1(b); *P* < 0.01), compared to the control cells. Interestingly, the addition of TGF-*β*3 together with HS results in significant increases in GAG synthesis (Figure 1(a); *P* < 0.01 at days 7 and 14 and *P* < 0.05 at day 21), cartilage-specific gene expression of SOX9 (*P* < 0.01), ACAN (*P* < 0.01), and COL2A1 (*P* < 0.05) (Figure 1(b)), and cartilage matrix-protein secretion (Figure 1(c)), as compared to the cells treated with TGF-*β*3 alone. These results show that HS enhances TGF-*β*3-induced chondrogenic differentiation of hMSCs.

### 3.2. HS Modulates the Expression Mode of TGF-*β* Receptors

**Figure 2:**
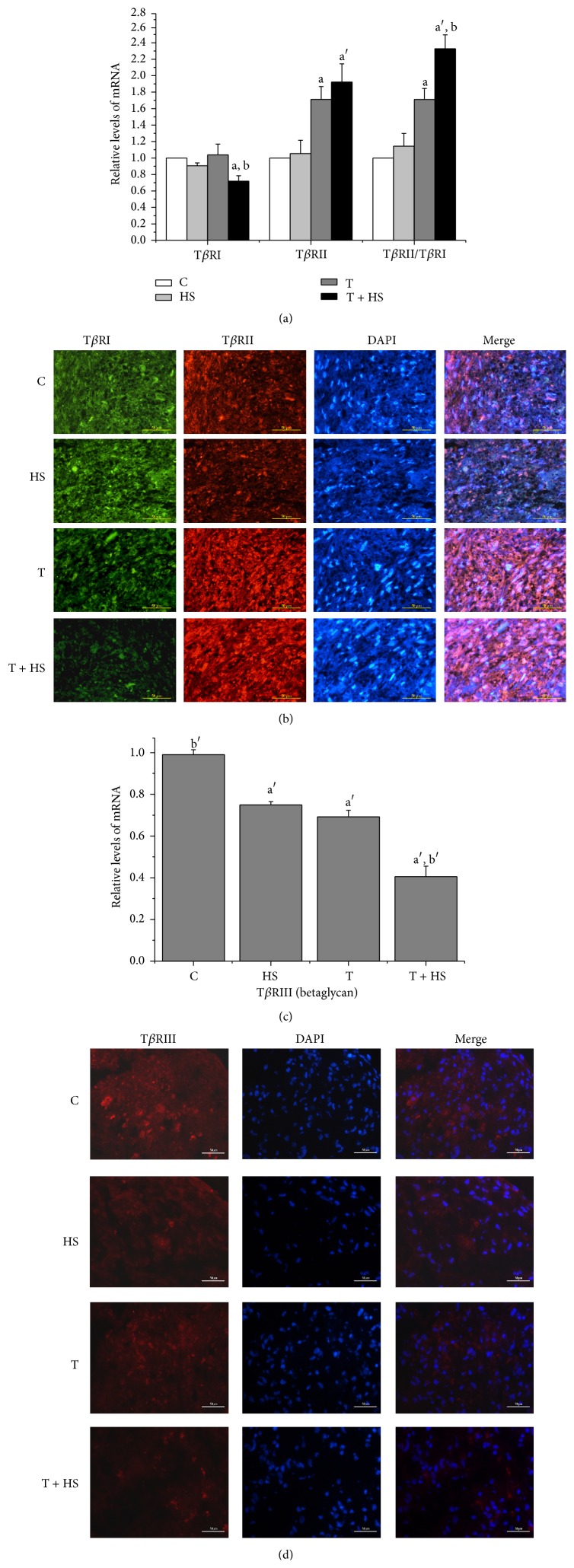
HS modulates expression mode of TGF-*β* receptors. Cells were cultured in four different media (C, HS, T, and T + HS) for 7 days. (*n* = 3) (a) mRNA expression of T*β*RI and T*β*RII and ratio of T*β*RII to T*β*RI by real-time RT-PCR. ^a^ *P* < 0.05 versus C group, ^a′^ *P* < 0.01 versus C group, and ^b^ *P* < 0.05 versus T group. (b) The expressions of T*β*RI and T*β*RII are visualized by immunofluorescence staining using anti-T*β*RI (green) and anti-T*β*RII (red) antibodies. Nuclei are counterstained using DAPI (blue). The far right panels show merged images. Scale bar = 50 *μ*m. (c) mRNA expression of T*β*RIII by real-time RT-PCR assay. ^a′^ *P* < 0.01 versus C group, ^b′^ *P* < 0.01 versus T group. (d) The expressions of T*β*RIII are visualized by immunofluorescence staining using anti-T*β*RIII (red) antibodies. Nuclei are counterstained using DAPI (blue). The far right panels show merged images. Scale bar = 50 *μ*m.

There was no difference between the T*β*RI mRNA levels and the protein expression of the cells treated with HS alone or TGF-*β*3 alone and those of the untreated control cultures (Figure 2(a); *P* > 0.05) (Figure 2(b)). However, the combination of TGF-*β*3/HS treatment decreased T*β*RI mRNA levels (Figure 2(a); *P* < 0.05) and T*β*RI protein expression (Figure 2(b)) compared to those of the other groups. The HS treatment alone did not increase the expression of the T*β*RII gene (Figure 2(a); *P* > 0.05) or the protein expression (Figure 2(b)) compared to that of the untreated control cultures. Both the TGF-*β*3 treatment alone and the combined TGF-*β*3/HS treatment enhanced the mRNA levels of the T*β*RII gene (Figure 2(a); *P* < 0.05) and T*β*RII expression (Figure 2(b)) compared to those of the control. There was no obvious difference in T*β*RII gene levels (Figure 2(a); *P* > 0.05) or T*β*RII expression (Figure 2(b)) between the TGF-*β*3 treatment alone and the combined TGF-*β*3/HS treatment. Analysis of the ratio of T*β*RII to T*β*RI levels revealed that the ratio of cells treated was higher in the TGF-*β*3-only treatment and the combined TGF-*β*3/HS treatment than in the controls (Figure 2(a); *P* < 0.05 and *P* < 0.01, resp.). T*β*RII/T*β*RI levels increased dramatically in the combined TGF-*β*3/HS treatment compared to those of the TGF-*β*3-only treatment (Figure 2(a); *P* < 0.05). For T*β*RIII, the HS alone or TGF-*β*3 alone treatment and the combination of TGF-*β*3/HS treatment decreased T*β*RIII mRNA levels (Figure 2(c); *P* < 0.01) and T*β*RIII protein expression (Figure 2(d)) compared to those of the control. T*β*RIII levels decreased obviously in the combined TGF-*β*3/HS treatment compared to those of the HS alone or TGF-*β*3 alone treatment (Figure 2(c); *P* < 0.01) (Figure 2(d)).

### 3.3. HS Strengthens TGF-*β*3-Mediated Phosphorylation of Smad2/3

**Figure 3:**
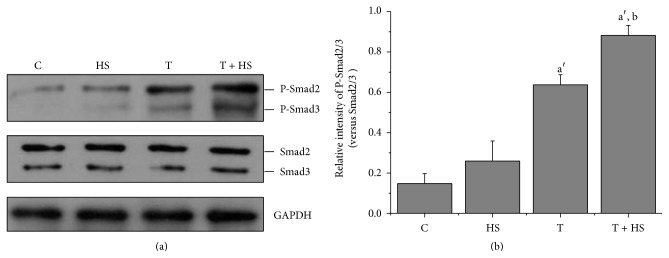
HS strengthens TGF-*β*3-mediated Smad2/3 phosphorylation. Cells were cultured in four different media (C, HS, T, and T + HS) and were harvested at 24 h. (a) Western blot for protein levels of P-Smad2/3, total Smad2/3, and GAPDH. (b) Quantification of protein levels of P-Smad2/3 normalized to total levels of Smad2/3. Error bars represent the means ± SD, *n* = 3. ^a′^ *P* < 0.01 versus C group, ^b^ *P* < 0.05 versus T group.

As shown in Figure 3, HS alone did not affect the expression of phospho-Smad2/3. However, both the TGF-*β*3-only treatment and the combined TGF-*β*3/HS treatment strongly activated the phosphorylation of Smad2/3. It is worth noting that HS further enhanced phospho-Smad2/3 activation induced by TGF-*β*3.

### 3.4. SB431542 Blocks HS-Activated TGF-*β*3-Mediated Phosphorylation of Smad2/3

**Figure 4:**
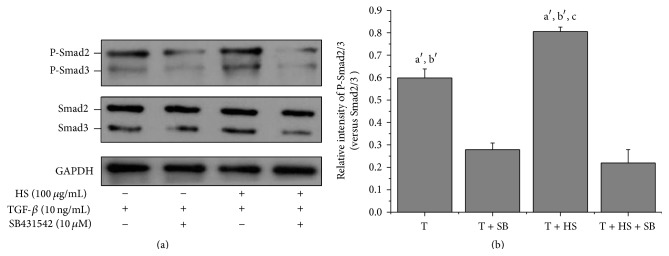
SB431542 blocks HS-activated TGF-*β*3-mediated Smad2/3 phosphorylation. Cells were cultured in control medium supplemented with TGF-*β*3 (T), SB431542 treated for 2 h before treatment with TGF-*β*3 (T + SB), TGF-*β*3 together with HS (T + HS), or SB431542 treated for 2 h before treatment with TGF-*β*3 together with HS (T + HS + SB). Samples were harvested at 24 h. (a) Western blot for protein levels of P-Smad2/3, total Smad2/3, and GAPDH. (b) Quantification of protein levels of P-Smad2/3 normalized to total levels of Smad2/3. Error bars represent the means ± SD, *n* = 3. ^a′^ *P* < 0.01 versus T + SB group, ^b′^ *P* < 0.01 versus T + HS + SB group, and ^c^ *P* < 0.05 versus T group.

SB431542 inhibited TGF-*β*3-activated Smad2/3 phosphorylation and completely inhibited HS-enhanced TGF-*β*3-activated Smad2/3 phosphorylation (Figure 4(a)). There was no statistical difference between the phosphor-Smad2/3 levels of the cells treated with TGF-*β*3 in the presence of SB431542 (T + SB) or in those treated with TGF-*β*3 and HS in the presence of SB431542 (T + HS + SB) (Figure 4(b); *P* > 0.05).

### 3.5. SB431542 Inhibits HS-Enhanced TGF-*β*3-Induced Chondrogenic Differentiation of hMSCs

**Figure 5:**
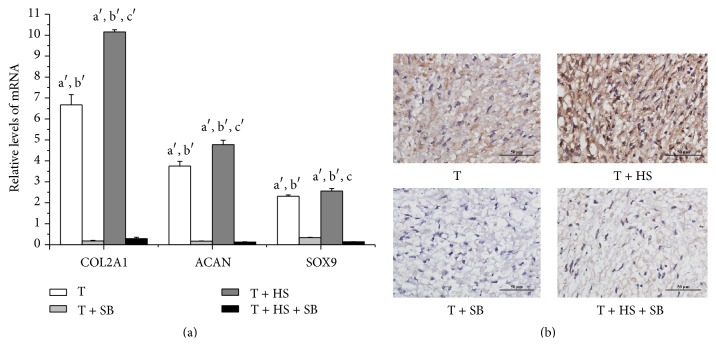
SB431542 inhibits HS-enhanced TGF-*β*3-induced chondrogenic differentiation of hMSCs. Cells were cultured in four different media (T, T + HS, T + HS, and T + HS + SB) for 7 days. (a) mRNA levels of SOX9, ACAN, and COL2A1 are measured by real-time PCR. Error bars represent the means ± SD, *n* = 4. ^a′^ *P* < 0.01 versus T + SB group, ^b′^ *P* < 0.01 versus T + HS + SB group, ^c^ *P* < 0.05 versus T group, and ^c′^ *P* < 0.01 versus T group. (b) Immunohistochemistry staining for collagen type II. *n* = 3, scale bar = 50 *μ*m.

The RT-PCR analysis showed that the combined TGF-*β*3/HS treatment significantly increased cartilage-specific gene (SOX9, ACAN, and COL2A1) expression when compared to that of the cells treated with TGF-*β*3 alone (Figure 5(a); *P* < 0.01) (Figure 1(b); *P* < 0.01). The expression of cartilage-specific genes decreased in the T + SB and T + HS + SB groups as compared to that of either the T or T + HS groups (Figure 5(a); *P* < 0.01). There was no statistically significant difference in the expression levels of SOX9, ACAN, and COL2A1 between the T + SB group and T + HS + SB group (Figure 5(b); *P* > 0.05). Immunohistochemistry for collagen type II showed similar results, with the expression of collagen type II that was enhanced by TGF-*β*3 completely inhibited by SB431542 (Figure 5(b)).

## 4. Discussion

HS-stimulated cartilage nodule formation and growth in micromass cultures of chick limb bud mesenchyme have been reported [26]. However, our results showed that exogenous HS alone did not strongly induce chondrogenesis of MSCs in vitro (Figure 1). The discord may be due to MSCs being more original compared to the cells derived from chick limb bud mesenchyme. Our results also confirmed the findings of other studies, which demonstrated that TGF-*β*3 induced chondrogenic differentiation of MSCs [7]. Interestingly, we found that HS significantly enhanced TGF-*β*3-induced chondrogenic differentiation and cartilage-specific gene expression of hMSCs (Figure 1). Fisher et al. reported that exogenous HS enhances the ability of bone morphogenetic protein 2 (BMP-2), another important member of the TGF-*β* superfamily, to promote chondrogenic differentiation in micromass cultures of limb mesenchymal cell [27]. Based on these studies, perlecan, a HSPG in the ECM, was complexed with collagen II to construct a biomimetic material. The resulting material was able to bind more BMP-2 than a type II collagen scaffold, leading to enhanced chondrogenic differentiation [28]. Our results further demonstrate the important role of HS in regulating the chondrogenic activity of the TGF-*β* superfamily. They also provide an experimental basis for HS or HSPG as biomimetic biomaterials or drug interacting with TGF-*β* for cartilage tissue engineering.

HS might regulate signaling-molecule response by modulating the interactions between growth factors with their receptors [27]. To explore the molecular mechanism by which HS enhances TGF-*β*3-induced chondrogenic differentiation and to determine whether HS increased TGF-*β* signaling, we observed the effect of HS on TGF-*β*3-mediated T*β*R (I, II, and III) expression and phosphorylation of Smad2/3. The results showed that HS modulated the TGF-*β*3-induced expression of TGF-*β* receptors, decreased T*β*RIII expression (Figure 2) but increased the ratio of T*β*RII to T*β*RI (Figure 2), and increased Smad2/3 phosphorylation (Figure 3). The results indicate that exogenous HS modulates the interactions between TGF-*β*3 with its receptors and activates downstream Smad2/3 signal pathway. A previous study reported that increasing the ratio of T*β*RII to T*β*RI in the TGF-*β* receptor/ligand complex provided a positive signal to augment TGF-*β*-induced cellular responses [10]. It is possible that HS increases the ratio of TGF-*β*3 binding to T*β*RII and T*β*RI and activates TGF-*β*/Smad2/3 signaling. There are two possible mechanisms in which exogenous HS modulates the interactions between TGF-*β*3 with its receptors. The binding of HS to TGF-*β*3 might directly facilitate the interaction of TGF-*β*3 with its receptors [29]. Alternately, exogenous HS inhibited endogenous HSPGs, T*β*RIII (betaglycan) expression (Figures 2(c) and 2(d)). It has been reported that T*β*RIII with larger HSPGs negatively modulates TGF-*β*-induced cellular responses by regulating the ratio of TGF-*β* binding to T*β*RII and T*β*RI [9, 10]. That may explain HS-induced promotion of TGF-*β*3-induced chondrogenic differentiation of MSCs.

Although Smad2/3 is a primary TGF-*β* signaling pathway for initiating chondrogenic differentiation, other pathways, such as the P38 pathway, are also activated by TGF-*β* during chondrogenesis [30]. Our study further showed that SB431542, a TGF-*β* signaling inhibitor, not only completely inhibited HS-stimulated, TGF-*β*3-mediated Smad2/3 phosphorylation (Figure 4) but also completely inhibited the effects of HS on TGF-*β*3-induced chondrogenic differentiation (Figure 5). These results demonstrated that TGF-*β*/Smad2/3 signaling is an exclusive and unique pathway, by which HS potentiates the TGF-*β*3-induced chondrogenic differentiation of MSCs.

## 5. Conclusions

This study demonstrated that exogenous HS enhanced TGF-*β*3-induced chondrogenic differentiation of hMSCs by facilitating interaction of TGF-*β*3 with its receptors and further activating downstream Smad2/3 signaling. These findings provide a potential strategy for the use of HS or HSPG as biomimetic biomaterials or drugs that cooperate with TGF-*β* for cartilage tissue engineering. Further research is required to explore the roles of T*β*RIII (betaglycan) in modulating TGF-*β*-induced chondrogenesis of MSCs by regulating the interaction of TGF-*β*3 with T*β*RII and T*β*RI. The corresponding experiments are in progress.

## Figures and Tables

**Table 1:** Primers used for real-time PCR.

Gene	Forward primer (5′ to 3′)	Reverse primer (5′ to 3′)
GAPDH	5′-AGAAAAACCTGCCAAATATGATGAC-3′	5′-TGGGTGTCGCTGTTGAAGTC-3′
Col2A1	5′-GGCAATAGCAGGTTCACGTACA-3′	5′-CGATAACAGTCTTGCCCCACTT-3′
ACAN	5′-TGCATTCCACGAAGCTAACCTT-3′	5′-GACGCCTCGCCTTCTTGAA-3′
SOX9	5′-AGCGAACGCACATCAAGAC-3′	5′-GCTGTAGTGTGGGAGGTTGAA-3′
T*β*RI	5′-ATTACCAACTGCCTTATTATGA-3′	5′-CATTACTCTCAAGGCTTCAC-3′
T*β*RII	5′-ATGGAGGCCCAGAAAGATG-3′	5′-GACTGCACCGTTGTTGTCAG-3′
T*β*RIII	5′-GTGTTCCCTCCAAAGTGCAAC-3′	5′-AGCTCGATGATGTGTACTTCCT-3′

GAPDH: glyceraldehyde-3-phosphate dehydrogenase; COL2A1: collagen type II; ACAN: aggrecan; SOX9: SRY (sex determining region Y)-box 9; T*β*RI/II/III: recombinant human transforming growth factor-*β* receptor type I/II/III.
